# Synovial Tissue: Turning the Page to Precision Medicine in Arthritis

**DOI:** 10.3389/fmed.2019.00046

**Published:** 2019-03-21

**Authors:** Clément Triaille, Bernard R. Lauwerys

**Affiliations:** ^1^Pôle de Pathologies Rhumatismales Systémiques et Inflammatoires, Institut de Recherches Expérimentales et Cliniques, Université catholique de Louvain, Brussels, Belgium; ^2^Department of Pediatric Haematology and Oncology, Cliniques Universitaires Saint-Luc, Brussels, Belgium; ^3^Department of Rheumatology, Cliniques Universitaires Saint-Luc, Brussels, Belgium

**Keywords:** synovial biopsies, rheumatoid arthritis, precision medicine, response to therapy, biologics

## Abstract

Rheumatoid arthritis (RA) is a chronic systemic inflammatory disease targeting the joints. Current treatment strategies are based on clinical, biological and radiological features, yet still fail to reach the goal of early low disease activity in a significant number of cases. Hence, there is a need for refining current treatment algorithms, using accurate markers of response to therapy. Because RA induces histological and molecular alterations in the synovium even before apparition of clinical symptoms, synovial biopsies are a promising tool in the search of such new biomarkers. Histological and molecular characteristics of RA synovitis are heterogeneous. Variations in synovial lining layer hyperplasia, in cellular infiltration of the sublining by immune cells of myeloid and lymphoid lineages, and in molecular triggers of these features are currently categorized using well-defined pathotypes: myeloid, lymphoid, fibroid and pauci-immune. Here, we first bring the plasticity of RA synovitis under scrutiny, i.e., how variations in synovial characteristics are associated with relevant clinical features (disease duration, disease activity, effects of therapies, disease severity). Primary response to a specific drug could be, at least theoretically, related to the representation of the molecular pathway targeted by the drug in the synovium. Alternatively, absence of primary response to a specific agent could be due to disease severity, i.e., overrepresentation of all synovial molecular pathways driving disease activity overwhelming the capacity of any drug to block them. Using this theoretical frame, we will highlight how the findings of previous studies trying to link response to therapy with synovial changes provide promising perspectives on bridging the gap to personalized medicine in RA.

## Introduction

Rheumatoid arthritis (RA) is a chronic inflammatory disease affecting mostly joints. RA diagnosis using the ACR/EULAR 2010 criteria relies on clinical and biological criteria (1, 2), resulting in early diagnosis and differentiation from other conditions. Yet, making a diagnosis of RA is not informative about the strong clinical heterogeneity that prevails regarding many aspects of the disease such as disease severity, development of erosions, functional impact, and last but not least, response to therapy. Several features at diagnosis are classically associated with more severe disease: elevated serum CRP, presence of anti-citrullinated peptides antibodies, x-ray erosions at baseline (3). Yet, these features perform poorly at the individual level and do not allow any accurate prediction regarding outcomes and response to treatment.

Treatment strategies in RA changed dramatically over the last decades. First, the development of biological or targeted synthetic Disease Modifying Anti-Rheumatic Drugs (b or tsDMARDS) provided physicians with new powerful targeted drugs. Second, the growing body of evidence underlining the importance of early disease control led to the current consensus on treat-to-target therapy. Yet, the current recommendations are built on a trial and error approach despite the inclusion of clinical, biological and radiological prognostic factors (4). Therefore, failure to achieve early low disease activity is not uncommon using the current treatment strategies (5, 6).

There is a strong rationale in refining treatment strategies in RA in order to tackle the heterogeneity in treatment responses and reach the goal of early disease control in a majority of patients. In a single patient perspective, the question is simple: what drug from our large arsenal should this particular patient receive to reach early disease control? Within a broader perspective, can rheumatology enter a new era of precision medicine?

Besides the clinical benefit urging us to choose the drug with the highest probability of resulting in low disease activity or remission, some other factors make this choice critical. First, preventing patient from exposition to inefficient, yet potentially toxic, medication is a must. In addition, one cannot overlook the economic considerations raised by these new drugs.

Analysis of synovial tissue in RA seems a promising approach to search for markers of disease severity and response to therapy. However, as opposed to other medical specialists, rheumatologists did not systematically harvest synovial biopsies in clinical practice, and their use long remained limited to research areas, despite the development of safe, non-invasive procedures (7–10).

As a consequence, the biology of RA synovitis did not unveil all its secrets, to say the least. In fact, out of the evidence available until now, it appears that heterogeneity is probably the most appropriate attribute to characterize RA synovitis, both from a histological and from a molecular point of view. Although the observation of such heterogeneous patterns holds promises in the search for correlations with heterogeneous clinical outcomes, our understanding of the in- or extrinsic factors driving the observed variations in synovial features is still limited, partly because most studies were performed on retrospective material, collected in small numbers of patients, resulting in significant methodological issues regarding patients' stratification. Despite these limitations, several intelligible patterns have emerged, which we will describe in the paragraphs below, with a particular focus on the use of gene expression profiling in RA synovitis in order to predict response to therapy.

## Synovial pathotypes in RA

RA is characterized by distinct changes in synovial architecture: proliferation of lining cells (macrophage and fibroblasts), proliferation of blood vessels in the sub-lining and infiltration by mononuclear cells (macrophages, T and B lymphocytes) (11). These changes are not specific to RA, but are also found in other rheumatic conditions, albeit with different amplitudes [e.g., higher grades of synovial hyperplasia or mononuclear cell infiltration in RA (12), increased hypervascularity in spondyloarthropathies (13, 14)]. Conversely, histological markers of synovitis vary significantly within the same condition. Ulfgren et al. reported back in 2000 that the degree of immune cell infiltration in RA synovitis can range from highly infiltrated to a low inflammatory pattern (15).

In 2014, Dennis et al. (16) introduced the concept of synovial pathotypes in RA according to the cellular and molecular composition of the synovium, and proposed a subdivision in 4 categories: lymphoid, myeloid, fibroid and pauci-immune. Thus, hierarchical clustering of microarray gene expression data led to the identification of these 4 subgroups of RA synovitis in a cohort of 49 patients based on gene expression profiles, and this corresponded to immunohistochemical evidence of T and B cell enrichment in the lymphoid subgroup, proportional enrichment of macrophages in the myeloid subgroup and a relative higher proportion of fibroblasts in the fibroid subgroup. Of interest was the increase in synovial myeloid scores (i.e., a quantitated evaluation of the overall expression of myeloid-associated transcripts in the synovium) in good-responders to TNF blockade, while lymphoid scores were equally distributed in non-, moderate-, or good-responders to these drugs.

Of note, the samples used in this study were obtained from RA patients with established disease undergoing arthroplasty or synoviectomy, treated with conventional synthetic or bDMARDs, and we will see below how these factors impact synovial features in RA. However, the concept that intrinsically distinct pathotypes underpin the organization of RA synovitis is a potential breakthrough, and deserves further discussion (17).

Identification of a lymphoid pattern characterized by a strong synovial enrichment in T and B cells is reminiscent of previous work related to the presence of lymphoid aggregates in RA synovitis. Ectopic lymphoid neogenesis occurs in 25% of RA synovial samples, and results in some cases in the formation of follicular dendritic cell-positive germinal centers (18–20). In previous studies, the presence of lymphoid aggregates was associated with disease severity, i.e., the risk of developing x-ray erosions (21). However, these results were not confirmed in later studies, performed on larger numbers of patients, in which no association was found between synovial lymphoid aggregates and clinical outcomes such as the development of erosions or increased disease activity (19, 22). In addition, these studies showed that lymphoid aggregates are also present in the synovium of other inflammatory diseases and correlated with the degree of overall synovial infiltration by inflammatory cells.

**Figure 1:**
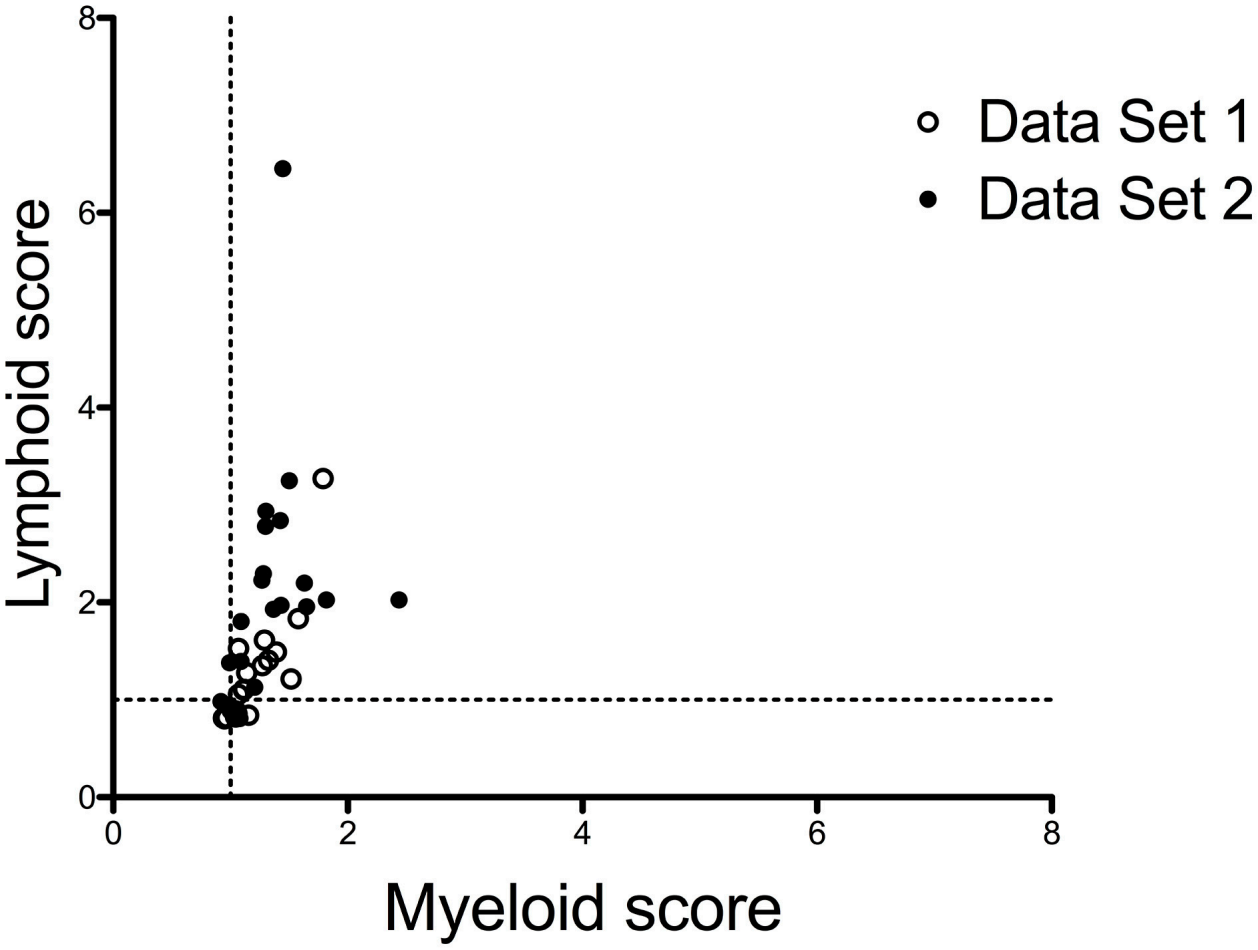
Correlations between myeloid and lymphoid scores in RA synovitis. Lymphoid and myeloid gene scores were calculated in 2 sets of 20 biopsies from patients with active RA, based on gene lists used by Dennis et al. (16), downloaded from https://arthritisresearch.biomedcentral.com/articles/10.1186/ar4555#MOESM3. Gene scores are the median values of the log2- transformed fold changes of each transcript belonging to the score compared to a reference group of 4 OA samples. Data set 1: synovial samples from RA patients with early disease, published in Ducreux et al. (23). Data Set 2: unpublished set of synovial samples, from RA patients with established disease. The characteristics of the patients in both data sets are displayed in Supplementary Table 1. Spearman correlation coefficient *r* = 0.7801.

Positive correlations between the presence of synovial lymphoid cells and overall synovial inflammation suggest that synovial lymphoid and myeloid scores might be inter-dependent, rather than mutually exclusive. Using the scores developed by Dennis et al. (16), we mined high-throughput transcriptomic data generated in two series of 20 RA biopsies, and found a strong correlation between both scores, with very few outliers displaying a preferential myeloid or lymphoid signature (Figure 1), indicating that activated myeloid and lymphoid cells in RA synovitis are part of a coordinated inflammatory response.

Yet, it remains plausible that differential activation of specific lymphoid cell subsets in RA synovitis is associated with relevant clinical outcomes. In this perspective, it should be stressed that transcriptomic studies performed on whole synovial biopsies might easily miss signals generated by rare cell populations, and do not always allow to differentiate between differential cell activation vs. representation across samples (24). The results of single synovial cells RNA sequencing studies (25) will obviously increase our ability to understand associations between specific synovial cell subsets and clinical phenotypes. For example, previous descriptive and functional approaches suggested an association between synovial B cell enrichment and early development of erosions in RA (26). Synovial B cells undergo affinity maturation and clonal selection in ectopic lymphoid structures (27, 28), especially in early disease. They locally produce ACPA (29) that have the known ability to activate osteoclasts (30). In addition, synovial B cells activate T cells (31), display an antigen-presenting cell phenotype (32), directly activate osteoclasts through production of RANKL (33), and are involved in the production of various cytokines (34).

Regarding high and low inflammatory synovitis, it is unclear how both patterns relate to each other. Two main hypotheses are currently proposed to explain this variability. First, intensity of synovial inflammation and disease activity could be linked. Second, high and low inflammatory synovitis could represent distinct entities driven by different physiopathological mechanisms. To address this issue, one main question arises: do high and low inflammatory synovitis differ in terms of clinical phenotype (disease activity) or biological mechanisms? In a recent study (35), Orr et al. used a semi-quantitative score of inflammation to evaluate synovial biopsies obtained from 189 RA patients. They showed a significant, albeit weak, correlation (*r* = 0.23) between inflammatory scores and DAS28-CRP. The correlation with serum CRP was significant as well, and stronger (*r* = 0.43), suggesting that synovial tissue infiltration by immune cells could be related to global disease activity.

By contrast, in a study performed on 39 synovial samples from patients with longstanding RA (36), global RNA sequencing results divided patients in 3 subgroups according to their gene expression profiles: high, medium and low inflammatory subtypes. Deconvolution algorithms indicated that the 3 subtypes displayed small but significant variation in terms of inferred immune cell subsets. Of note, the 3 subtypes differed in markers of systemic inflammation (CRP, ESR) but not clinical markers (swollen joint count, tender joint count) and treatment, and it is therefore unclear whether the level of synovial inflammation was or not an independent variable in this group of samples.

Finally, in another study (37), Kasperkovitz et al. studied gene expression profiles in both whole synovial biopsies and cultured fibroblast-like synovial cells (FLS) from 10 RA patients. Intriguingly, they found that cultured FLS from high and low inflammatory synovitis kept distinct gene expression profiles *in vitro*, thereby suggesting that the differences between these conditions could be driven internally by a stable phenotypical trait in non-autoimmune synovial cells.

Additional work is needed in order to assess whether synovial pathotypes, in particular low- vs. high-inflammatory synovitis, are associated with different underlying pathogenic mechanisms, hence require differential diagnostic and therapeutic approaches. From the evidence accumulated thus far, synovial phenotypes display a high level of plasticity. As expected, extrinsic factors, in particular disease activity, display a significant influence on synovial phenotype, and this is further illustrated in the following paragraphs describing variations in synovial gene expression profiles in different clinical situations. Yet, evidence suggests that a pauci-immune, by opposition to a high-inflammatory, pathotype is found in RA synovial biopsies as an intrinsic presentation of the disease, independently of disease activity. How this observation translates in clinically relevant decisions further needs to be evaluated.

## Factors Contributing to Synovial Heterogeneity in RA

### Diagnosis and Stage

Not surprisingly, gene expression profiles in the synovium are dependent on the underlying disorder. Thus, Nzeusseu Toukap et al. compared gene expression patterns in synovial biopsies from patients with systemic lupus erythematosus (SLE), RA, osteoarthritis (OA), psoriatic arthritis and gout (12). SLE biopsies were characterized by the spontaneous overexpression of interferon-induced genes. RA biopsies had a typical lymphoid signature (overexpression of T- and B cell activation-associated transcripts) and OA samples were characterized by the overexpression of transcripts associated with extracellular matrix turnover. Because it is a hallmark of synovial inflammation, a dominant myeloid signature was not found in any of these conditions, thereby also demonstrating how the choice of the comparator impacts the results of *ex vivo* studies.

Patients with longstanding RA display joint modifications associated with secondary or primary OA, which probably impact the results of synovial gene expression profiling experiments, although the evidence is scarce, and not always concordant. Thus, comparison of 10 patients with end-stage destructive disease undergoing joint replacement to 13 RA patients also with established disease, but active synovitis showed higher numbers of macrophages in the lining and sublining of patients with active synovitis, whereas differences in B and T cells were not significant (38). By contrast, Baeten et al. did not evidence any difference in histological features and immune cell proportions between early and longstanding RA synovial samples (13). Using 10,000 probes cDNA microarrays, Lequerré et al. compared gene expression profiles between 4 early and 4 longstanding RA synovial samples (39). Early RA synovitis was enriched in transcripts involved in the following processes: immunity and host defenses, stress responses, T cell-mediated immunity, and tumor suppressor and major histocompatibility complex (MHC) class II-mediated immunity whereas longstanding RA was enriched in cell cycle, cell surface receptor-mediated signal transduction, cell cycle control, ligand-mediated signaling, apoptosis inhibition, and granulocyte-mediated immunity. By contrast, Tsubaki et al., using 23,040-probes cDNA microarrays, studied synovial biopsies from early (*n* = 12) vs. longstanding (*n* = 4) RA undergoing arthroplasty, and did not evidence significant transcriptomic and histological differences between both groups (40). Overall, these results are compatible with the presence of a lower inflammatory load in longstanding RA synovitis. However, they also demonstrate how complex the interpretation of synovial biopsy studies might be when performed on low numbers of samples or retrospective material, in which interfering variables such as disease activity, ACPA status, or therapies potentially play a confounding role.

Whether patients with undifferentiated arthritis (UA) display RA-like synovial gene expression patterns before they progress to full-blown RA is also a question requiring large prospective studies to be addressed properly. Using a set of 100 transcripts, based on their ability to discriminate RA from other inflammatory disorders in synovial tissue, we found that an accurate diagnosis of RA could be predicted in UA patients only when a combination of synovial transcriptomic and clinical data were combined, in line with the hypothesis that synovial samples rather display an undifferentiated synovial gene expression pattern when they originate from UA patients (41).

### ACPA Status

van Oosterhout et al. compared synovial histological and immunohistological features in 34 ACPA+ vs. 23 ACPA– RA patients with established disease (average disease duration: 9.2 years). Expression of CD3 and CD8 was significantly higher in ACPA+ compared to ACPA– patients, while there was no difference in expression of CD4, CD19, or CD68. Semi-quantitative evaluation of synovial lining layer thickness and synovial fibrosis were higher in ACPA– patients (42). Similarly, Orr et al. compared synovial biopsies from 78 ACPA+ vs. 45 ACPA– patients, and found increased expression of CD3, CD8, and CD19 and more B cell aggregates in ACPA+ compared to ACPA patients (but not CD4 nor CD68) (26). In both cases however, disease activity scores were significantly higher in ACPA+ patients, which introduced a potential bias in the analyses, underscoring again the need for extensive patients' stratification in synovial biopsy studies.

### Clinical Disease Activity

We discussed previously the link between disease activity and histological signs of inflammation in synovial tissue. Not surprisingly, variations in disease activity also translate in variations in transcriptomic signatures observed in synovial biopsies from RA patients. van Baarsen et al. studied 17 RA synovial biopsy samples (43). Unsupervised hierarchical clustering divided them in 2 groups characterized by high vs. low inflammatory molecular signatures. The high inflammatory group was enriched in transcripts involved in the following biological processes: T-cell mediated immunity, cytokine- and chemokine-mediated signaling pathway and B-cell- and antibody-mediated immunity. Conversely, the low inflammatory group overexpressed genes associated with developmental processes, ectoderm development, and signal transduction. Disease activity (DAS28, TJC, ESR, CRP) was higher and disease duration was shorter in the high inflammatory group.

We looked at the link between synovial transcriptomic profiles and disease activity (DAS28CRP, CDAI, SDAI) in a series of 65 RA synovial biopsies (44) and found a strong correlation between all 3 measures and transcripts associated with an overwhelming lymphoid, but also, to a lesser extent, myeloid (TNFα-dependent) signature. Of note, the samples used in this study were obtained from untreated patients, but also from patients treated with methotrexate, tocilizumab and rituximab, drugs that preferentially down-regulate lymphoid transcripts in RA synovitis (see below). Because these drugs also decrease disease activity, correlations between disease activity and gene expression patterns using such samples necessarily increase the weight of lymphoid transcripts. Restricting the analyses to the 21 samples obtained from untreated patients restored the balance between lymphoid- and myeloid-associated transcripts in the correlation study with disease activity. These results point to an important link between clinical disease activity and synovial molecular signatures, thereby opening stimulating questions about the mechanisms driving disease activity in RA. Clinical disease activity measures the global burden of disease, and is based on the integration of systemic variables: number of tender/swollen joints, acute phase reactants and patient's, sometimes physician's, assessment of global disease activity. The meaning of the link between such global measures and gene expression profiles in a single joint remains to be elucidated. Is synovial gene expression the reflection of a disseminated systemic inflammation or is systemic disease activity driven by locally-initiated inflammatory processes? Finally, these results also demonstrate how clinical parameters (in this case therapies) affect the results of synovial gene expression studies, hence need to be tightly controlled.

### Effect of Therapies

RA drugs display significant effects on synovial cell populations and transcriptomic profiles, as evidenced by several longitudinal studies in which synovial biopsies were collected prospectively before and after administration of therapy. These results were determining in the identification of synovial molecular pathways correlating with response to therapy, and contributed to a better understanding of the mechanisms driving synovial inflammation. They also opened new perspectives in terms of personalized medicine and prediction of response to therapy, as discussed below.

Immunohistochemistry studies showed differences in cell populations before and after administration of effective drugs. As expected, proportions of all infiltrating inflammatory cells decreased in response to therapy, although the amplitude of the changes observed in specific cell populations were different according to the modes of action of the drugs. Thus, 3 months after initiation of tocilizumab (an anti-IL6R antibody) therapy in early RA patients (23), a relatively stronger decrease in infiltrating CD3 positive T cells was observed compared to other cell types, in line with the known T cell growth factor properties of IL6. By contrast, adalimumab (a TNFα-blocking antibody) displayed relatively stronger effects on proportions of CD68 positive cells compared to other synovial cell populations, 3 months after administration of the drug to methotrexate-resistant RA patients (45). We also investigated the effects of rituximab (anti-CD20 antibody) therapy on synovial cell populations before and 3 months after therapy (46). We found that B cells were depleted in the majority (18/20) of the samples, but the drug also displayed a significant effect on IL17 producing T cells (47), thereby supporting the hypothesis that B cells also play a role as antigen-presenting cells in RA synovitis. Differential responses of synovial cell populations to rituximab vs. TNF-blocking agents is also apparent from observations reported by other groups (48–51).

Interestingly, Bresnihan et al. (52, 53) found a correlation between the overall effects of several drugs (prednisolone, methotrexate, gold salts, leflunomide, infliximab, and rituximab) on disease activity in groups of patients and the decrease in CD68 positive macrophages in the sublining: the stronger the overall decrease in disease activity, the stronger the decrease in sublining CD68 positive cells in response to a given drug. It is not clear whether the association holds at the individual level, but these observations are of interest from a pathogenic point of view, as they support the role of synovial macrophages as a common mediator of disease activity in RA.

In several studies, the global molecular effects of therapies in RA synovitis were also investigated. Most of these studies were performed on low numbers of patients, yet delivered interesting clues on the modes of actions of these drugs. Lindberg et al. (54) performed transcriptomic studies on synovial biopsies from 10 RA patients before and 9 weeks after administration of infliximab therapy. A positive TNFα stain was detected at baseline in 4 of them, in whom the infliximab-induced molecular changes were the most striking, i.e., differential expression of 1,058 transcripts involved in immune responses, cell communication, signal transduction and chemotaxis. Similar patterns of differential gene expression were found in the subgroup of patients who were good-responders.

Our group carried out gene expression profiling of synovial biopsies from 12 patients before and 3 months after adalimumab therapy (45). In good responders (*n* = 6), adalimumab induced the down-regulation of 632 transcripts that were mainly involved in regulation of inflammatory responses (production of chemokines and cytokines) and cell division.

Both studies showed a good overlap of the biological themes affected by TNF inhibitors in RA synovitis. By contrast, we observed very different results when the molecular effects of other drugs were evaluated. Thus, we performed pathway analyses of differentially expressed genes in the synovium of TNF inhibitor-resistant patients prior to and 3 months administration of rituximab therapy (46) (*n* = 12 patients). In these samples, rituximab induced the downregulation of transcripts involved in immune responses, chemotaxis, T cell activation and immunoglobulins. In addition, rituximab led to upregulation of genes involved in wound healing. A similar study (23) was performed on synovial biopsies obtained from 12 early RA patients before and 3 months after administration of tocilizumab therapy. Pathway analyses of differentially expressed indicated a downregulation of transcripts involved in T cell activation and chemokines and an induction of transcripts involved in healing processes. A very similar pattern of molecular changes was observed in the synovium of early RA patients in response to methotrexate therapy, although the amplitude of the effects was lower.

When comparing the transcriptomic effects of the drugs reported in the previous paragraphs, we found a striking overlap between the effects of tocilizumab, rituximab and methotrexate (23). A transcript down- or upregulated by one of these drugs had a high probability to be similarly up- or downregulated by another drug. By contrast, there was no common ground between the molecular effects of adalimumab and the three other drugs. These observations lend further support to the concept that molecular pathways in RA synovitis are built along two axes: a lymphoid axis (T cell activation, chemokines) targeted by drugs such as tocilizumab, rituximab or methotrexate, and a myeloid axis (inflammation, cell division) targeted by TNF blocking agents.

It is important to keep in mind that changes in transcriptomic profiles in RA synovitis in these studies are influenced by changes in cell populations before and after administration of the drug. The results of single cell RNA sequencing studies will make it easier to understand how specific drugs interfere with dysregulated cellular pathways in the disease. This is for example the case regarding the induction of transcripts involved in wound healing pathways in response to several drugs (23, 46), an effect in which increased representation of resident cells (following a decrease in the presence of inflammatory cells) could play a role. Nevertheless, such description of the global effects of these drugs in the synovium provided clinicians and researchers with unique tools, leading to new research hypotheses on e.g., potential drug interactions in the treatment of RA (drugs that do not share the same molecular effects might have additive or synergistic effects) and response to therapy.

### Gene Expression Profiling in RA Synovitis and Prediction of Response to Therapy

Response to therapy in RA follows a prototypical pattern, common to all drugs used in the disease: 30–40% good response, 30–40% moderate response, and 30–40% poor response (6). Response to therapy is usually assessed after 3 months (55) (although some drugs, such as methotrexate, may still improve their therapeutic effect after a longer period of time). However, in the context of a treat-to-target strategy aiming at achieving early remission, clinical care of RA patients would be improved if response to therapy could be predicted prior to initiation of therapy, using accurate markers.

Response to therapy as such is a complex phenotype. Whether ACR or EULAR response criteria (56, 57) are used, response to therapy is a composite score that integrates changes in objective (acute phase reactants, swollen and tender joint counts) and more subjective (patient's or physician's global assessment of disease activity) variables that do not necessarily overlap. For example, searching for clinical measures correlating with the expression of TNFα-induced transcripts in RA synovitis (44), we found a very poor correlation with patient's global assessment of disease activity, while the best correlation was found with physician's global assessment, indicating that both variables do not reflect similar features. It is therefore important to keep in mind that response to therapy is not a homogeneous variable, which strongly affects the ability to predict it accurately.

Theoretically, absence of adequate clinical response to a specific drug could be due to synovial underrepresentation of the pathway targeted by the drug. Alternatively, disease severity could drive poor response to therapy, through synovial overexpression of several (if not all) molecular pathways driving disease activity in the synovium, overwhelming the capacity of the drug to inhibit any of them (a situation that could characterize patients failing one drug after the other). Finally, secondary loss of response to therapy due to the development of anti-drug antibodies (58, 59) is a situation very different from primary lack of response, which is discussed in the following paragraphs.

Several studies were performed in order to find associations between synovial molecular patterns at baseline and response to several drugs, in particular TNF-blocking agents (Table 1). In 2008, Van der Pouw Kraan et al. (61) compared baseline synovial gene expression profiles in 12 responders (defined based on a DAS28 reduction>or = 1.2 at 4 months) vs. 6 non-responders to infliximab therapy. Molecular pathways upregulated in responders included: T-cell mediated immunity, cell surface receptor mediated transduction, MHCII mediated immunity, cell adhesion, cytokine and chemokine mediated signaling pathway, cell adhesion mediated pathway, signal transduction, macrophage mediated immunity. In 2010, Lindberg et al. (54) obtained synovial biopsies from 62 methotrexate-resistant patients prior to initiation of infliximab therapy, and compared gene expression profiles in 18 good- vs. 14 poor-responders according to EULAR response criteria. At first, they also found a slight enrichment in transcripts involved in chemotaxis, inflammatory responses and leukocyte activation in good-responders. However, they observed that these molecular patterns were rather associated with the presence of lymphoid aggregates in synovial tissue, found more often in good-responders in this group of patients. It was however unclear whether the overrepresentation of lymphoid aggregates positive patients in good responders was a confounding factor or reflected a real biological difference. After stratification of the samples for the presence and size of lymphoid aggregates, they could not observe any robust gene expression differences between good- and poor-responders to the drug. In this context, it is noteworthy that presence of synovial lymphoid aggregates was associated with a better response to infliximab in histological study on 97 methotrexate-resistant RA patients (63).

**Table 1:** Studies using high-throughput gene expression profiling of the synovium to identify predictive markers of response to treatment.

**Reference**	**Treatment**	**Disease duration**	**Number of patients and response**	**Pretreatment disease activity score across groups**	**Response to treatment criteria**	**Ongoing DMARD or steroid treatment**	**Histology**	**Main findings**	**Platform**	**Transcriptomic data available**	**Sampling technique**
Lindberg et al. (60)	IFX	0.6–26 years	GR = 3 MR = 5 NR = 2	N/A	EULAR response criteria	MTX: 10/10 Pred (<7.5 mg/j): 5/10	N/A	Up regulated pathways in GR: Immune response, cell communication, chemotaxis, signal transduction	KTH microarray	Array express	Arthroscopy
van der Pouw Kraan et al. (61)	IFX	0.6–26 years	R = 12 NR = 6	DAS R: 6.0 (4.7-7.7) NR : 5.3 (3.7-7.2) CRP(mg/dl): R: 28(3–110) NR: 13 (5–49)	1.2 decrease in DAS28 score is response	MTX: 18/18 Pred: 5/18	CD3 and CD163 higher in R	Upregulated pathways in R: Immunity and defense, T-cell mediated immunity, Signal transduction, MHCII-mediated immunity	43 k human cDNA micro array (Stanford University)	Stanford micro array database	Arthroscopy
Badot et al. (45)	ADA	1–36 years (mean 10 years)	R = 20 NR = 5	DAS28-CRP (±SEM) R: 5.289 (±0.213) NR:4.774 (±0.186)	EULAR response criteria	MTX (23/25), Lefl (2/25) Pred (18/25)	CD3, CD15, CD20, CD68 staining showed no difference between R and NR	Genes overexpressed in NR vs. R are implicated in cell division and regulation of immune response and are induced by TNF-α	GeneChip HG u133Plus 2.0 array, Affymetrix	GSE15602	Needle arthroscopy
Lindberg et al. (54)	IFX	Mean(±SD) : 11 years (±9.5)	R = 48 NR = 14	DAS28 (±SD) R: 6 (± 0.9) NR 5.9 (±0.9) CRP (mg/dl) (±SD) R: 17 (±16) NR 14 (±18)	EULAR response criteria	MTX (62/62) Pred (16/62)	Over representation of lymphoid aggregates in GR	Presence of aggregates dominates the gene expression profile. GR group is enriched with aggregates positive patients	In-house array 17972 genes (KTH micro array)	GSE21537	Needle arthroscopy
Hogan et al. (62)	RTX	>1 year	R = 10 NR = 10	N/A	EULAR response criteria	MTX (18/20), Prednisone (11/20)	N/A	Higher expression of macrophages and T-cell related genes in R Higher expression of Interferon-α in NR	Home made qPCR panel (125 genes)	N/A	Arthroscopy
Dennis et al. (16)	TNFi/TCZ	See Lindberg et al. (54)	Pathotypes and corresponding gene scores were computed on 69 treated patients with >3 years disease duration. Patients from Lindberg et al. (54) were used for prediction of treatment response R = 48 NR = 14	See Lindberg et al. (54)	EULAR response criteria	See Lindberg et al. (54)	Immune cells subsets correlate with transcriptomic-defined clusters in patients used for pathotype determination	Myeloid Score at baseline is higher in GR vs. NR to IFX Identification of circulating biomarker associated with better response to TNFi or TCZ in ADACTA trial patients	GeneChip HG u133Plus 2.0 array, Affymetrix	GSE48780	Joint replacement or surgical synovectomy
De Groof et al. (44)	MTX or TCZ	Mean (±SD) 0.5 year (± 1.1)	GR = 9 MR = 7 NR = 5	DAS28CRP MTX group: 4.51 ± 1.49 TCZ group: 4.5 ± 1.1	EULAR response criteria	Untreated	N/A	Genes over expressed in NR are TNF-α and IL6-dependent	GeneChip HG u133Plus 2.0 array, Affymetrix	GSE45867	Needle arthroscopy

*GR, Good responders; MR, moderate responders; NR, non-responders; R, responders; MTX, Methotrexate; Lefl, Leflunomid; Pred, Prednisolone; RTX, Rituximab; TCZ, tocilizumab; TNFi, TNF-inhibitor; IFX, Infliximab; ADA, Adalimumab; ANA, anakinra*.

As discussed previously, Dennis et al. (16) mined a set of transcriptomic data generated from 49 synovial biopsies before administration of infliximab therapy. They found a higher myeloid score in good- compared to moderate- or non-responders to infliximab therapy, while lymphoid scores were not different across the three groups. Interestingly, they further investigated the concept by measuring serum biomarkers correlating with synovial myeloid (sICAM1) or lymphoid (CXCL13) scores in an additional cohort of 198 patients prior to administration of tocilizumab vs. adalimumab [ADACTA trial (64)]. In line with their synovial transcriptomic data, they found a positive association between the relative levels of both serum markers and response to infliximab or tocilizumab. Thus, high ICAM1/low CXCL13 patients had a 42% probability of reaching a ACR50 response to adalimumab vs. 13% in low ICAM1/high CXCL13 patients. Conversely, 69% low ICAM1/high CXCL13 patients reached a ACR50 response after administration of tocilizumab vs. 20% of the high ICAM1/low CXCL13 patients. Overall, the accuracy of these serum markers in predicting response to therapy was rather low. However, the experimental approach adopted by the authors was highly rational, and highlighted how synovial biopsy studies could lead to the development of useful biomarkers in daily clinical practice.

Using synovial biopsies (45) obtained at baseline in 25 methotrexate-resistant RA patients prior to administration of adalimumab therapy, we reported contrasting results in comparison to the previous observations. Thus, we found that transcripts overexpressed in poor-responders to adalimumab therapy were induced by TNFα itself or by IL1β (or by a combination of both cytokines) in cultured FLS, and confirmed these results by immunohistochemistry on the same samples. IL7R was one of the transcripts most overexpressed in poor-responders, and this observation led us to identify how the soluble form of the receptor (sIL7R) is produced by stromal cells in response to inflammatory cytokines and secreted in the serum. Hence, serum sIL7R measurements are a marker of tissue (instead of systemic) inflammation in RA, high serum concentrations being indicative of high concentrations of inflammatory cytokines (TNFα, IL1β) in the synovium. We measured sIL7R serum concentrations in sera from DMARD-resistant RA patients prior to initiation of infliximab therapy (65), and found significantly higher concentrations in 18 poor- compared to 57 good- and moderate-responders (predictive positive and predictive negative values in predicting response to therapy were 87 and 71%, respectively). These observations support the hypothesis that non-response to TNF blockade in these two groups of RA patients was driven by higher disease severity, resulting in higher synovial expression of pro-inflammatory cytokines, not adequately blocked by the administered antibodies. These results are not necessarily in contradiction with the ones reported in the previous paragraphs, but they do not intuitively point at the same concepts. In one case, response to TNF blocking agents requires the presence of a TNF signature to be blocked. In the other case, too much synovial impregnation in TNFα opposes the effect of TNF blocking agents. If anything, these results demonstrate the need for large scale prospective trials on large numbers of patients, in order to reconcile all observations and identify accurate markers of response to TNF blocking agents in RA.

We also performed transcriptomic and immunohistochemistry studies in synovial biopsies obtained in early RA patients (44), in order to identify markers of poor response to first line therapies. GADD45B stains (GADD45B is induced in macrophages upon stimulation with TNFα) were carried out in synovial samples obtained in two different groups (*n* = 46 and *n* = 35) of patients before initiation of first-line therapies, in particular methotrexate (*n* = 17 and *n* = 35). We observed that GADD45B expression in synovial tissue was significantly higher in non-responders to methotrexate or to any first line therapy in both groups, thereby supporting the hypothesis that higher levels of synovial inflammatory cytokines drive poor response to any therapy in RA, in line with the concept linking higher disease severity and treatment responses.

In 2012, Hogan et al. (62) performed qPCR studies on synovial biopsies from 20 RA patients resistant to TNF blockade prior to administration of rituximab therapy in order to study correlations between gene expression at baseline and short- (3 months) or long-term (21 months) response to therapy. When computed in a gene score, 16 out of the 125 genes tested were found to correlate with decrease in DAS scores over time. Genes associated with response to therapy were mostly involved in macrophage- and T-cell biology whereas genes associated with absence of response were involved in bone remodeling and IFNα response. The link between IFN signature and poor response to rituximab in RA is still elusive. It was however confirmed by studies performed on PBMC from patients with RA, in which a similar association between type 1 IFN signature and poor-response to the drug was found (66, 67).

## Conclusion

Synovial tissue in RA is heterogeneous from a cellular and molecular point of view. However, it seems that at least some part of this heterogeneity is explained by clinically relevant variables such as disease duration, disease activity and effects of therapies. Although numerous therapies are available to treat RA, it appears from their molecular effects in synovial tissue that they mainly target two major pathways in the synovium, associated with activation of myeloid vs. lymphoid cells. Whether these pathways define distinct RA subgroups or pathotypes, in addition to a low inflammatory pathotype, or whether RA synovitis is a molecular and cellular continuum, is a pending issue. It is also unclear at this stage whether enrichment of selected pathways in the synovium is associated with a preferential response to selected drugs. In some studies, evidence also links absence of response to therapy to RA drugs with the presence of markers of disease severity in the synovium rather than markers of response to a specific agent.

Most of the studies addressing these questions were performed on small number of samples. Comparing studies is a difficult endeavor not only because of the heterogeneity of the analytical techniques used by different groups, but even more because of the heterogeneity of the patients' populations included in these trials, in terms of disease duration, exposure to previous therapeutic agents, disease activity, and disease severity, all variables known to influence molecular patterns in synovitis.

Large-scale, multi-centric trials are ongoing, and will undoubtedly cast new lights on many uncertain matters raised in this review article. Homogenization of the pre-processing and analytical steps, in addition to international agreements on minimal reporting requirements in synovial biopsy studies are also highly expected outcomes of ongoing multi-centric initiatives in the field (68, 69). Finally, technological [single cell RNA sequencing (70)] and analytical [machine learning (36)] developments will undoubtedly enable us to capture meaningful patterns in RA synovial tissue, in order to help clinicians tailor their clinical decisions according to the individual characteristics of their patients.

## Data Availability

Publicly available datasets were analyzed in this study. This data can be found here: https://www.ncbi.nlm.nih.gov/geo/query/acc.cgi?acc=GSE45867.

## Author Contributions

All authors listed have made a substantial, direct and intellectual contribution to the work, and approved it for publication.

### Conflict of Interest Statement

BL holds shares (<€15,000) of DNALytics. The reviewer AM declared a past co-authorship with one of the authors BL to the handling Editor. The remaining author declares that the research was conducted in the absence of any commercial or financial relationships that could be construed as a potential conflict of interest.
