# Supplementary material for: Synovial Tissue: Turning the Page to Precision Medicine in Arthritis
Source: Front Med (Lausanne). 2019 Mar 21;6:46. doi: 10.3389/fmed.2019.00046 (PMC6437087; doi:10.3389/fmed.2019.00046)
Supplement: Supplementary file 1 [file Table_1.DOCX]

| **Clinical and biological characteristics of the patients included in the figure** | **Data Set 1** | **Data Set 2** |
| --- | --- | --- |
| Age (mean ± SD) | 52 ± 13 years | 54 ± 14 years |
| Disease duration (mean ± SD) | 0.3 ± 0.6 years | 10 ± 11 years |
| Females (%) | 70 | 100 |
| Ongoing treatment (%) |  |  |
| cDMARDS | 0 | 40 |
| bDMARDS | 0 | 40 |
| Prednisolone | 0 | 40 |
| No DMARDS | 100 | 20 |
| Erosive disease (%) | 30 | 100 |
| ACPA positive (%) | 70 | 90 |
| DAS28CRP (mean ± SD) | 4.40 ± 1.19 | 5.44 ± 1.08 |
| CDAI (mean ± SD) | 15.5 ± 9.2 | 29.0 ± 11.3 |
| SDAI (mean ± SD) | 17.4 ± 10.5 | 30.5 ± 12.4 |
| CRP (mg/L) (mean ± SD) | 19.1 ± 19.7 | 12.0 ± 24.5 |

**SUPPLEMENTARY TABLE 1 : characteristics of the patients included in the Figure.**
